# Knowledge, Attitudes, and Beliefs About Opioid Use Disorder Treatment in Primary Care

**DOI:** 10.1001/jamanetworkopen.2024.19094

**Published:** 2024-06-28

**Authors:** Brandon del Pozo, Ju Nyeong Park, Bruce G. Taylor, Sarah E. Wakeman, Lori Ducharme, Harold A. Pollack, Josiah D. Rich

**Affiliations:** 1 Rhode Island Hospital, The Warren Alpert Medical School of Brown University, Brown University School of Public Health, Providence, Rhode Island; 2 National Opinion Research Center, University of Chicago, Chicago, Illinois; 3 Massachusetts General Hospital, Harvard Medical School, Boston; 4 Services Research Branch, National Institute on Drug Abuse, North Bethesda, Maryland; 5 Crown Family School of Social Work, Policy, and Practice, University of Chicago, Chicago, Illinois; 6 The Miriam Hospital, The Warren Alpert Medical School of Brown University, Providence, Rhode Island

## Abstract

This survey study assesses the US public’s perception and awareness regarding medication for opioid use disorder and its availability in primary care settings.

## Introduction

Primary care physicians (PCPs), a frequent, convenient, nonstigmatized touchpoint with health care, hold promise for expanding access to buprenorphine, the only office-based medication for opioid use disorder (MOUD) with demonstrated effectiveness in reducing overdose mortality.^1^ To expand buprenorphine-based treatment, federal policy has eliminated specialized training requirements for prescribing physicians and lifted patient caps. A wide gap remains, however, between buprenorphine need and provision in primary care settings.^2^

Research has explored barriers and facilitators of MOUD from physicians’ perspectives.^3^ While evidence suggests patients may respond favorably to MOUD in primary care settings,^4,5^ we know little of their awareness that MOUD can be prescribed there or opinion of whether PCPs ought to provide MOUD, whether patients would seek it, and whether they would refer someone to their PCP to obtain it. This survey study explored these questions.

## Methods

Data were obtained from a June 2023 cross-sectional survey for the Justice Community Opioid Innovation Network. The survey was administered in English and Spanish and offered online and by telephone to a probability-based, nationally representative sample of adults 18 years or older drawn from the National Opinion Research Center’s (NORC) AmeriSpeak panel. The NORC Institutional Review Board deemed this study exempt from review. Consent was obtained orally from telephone participants and electronically from online participants. We followed the AAPOR reporting guideline.

Analyses were performed with Stata 15.0 (StataCorp). Weighted Pearson χ^2^ tests and ad hoc regression were used, with prespecified 2-tailed significance of *P* < .05. Survey items and weighting methods are presented in the eAppendix in Supplement 1. Race and ethnicity data were collected to examine the relevant demographic differences in participant responses.

## Results

A total of 1234 individuals responded (700 females [56.5%], 539 males [43.5%]; 11.5% identifying as Black, 15.2% as Hispanic or Latino, 68.4% as White, and 4.8% as other or with ≥2 race and ethnicity; ages were reported categorically to protect confidentiality) (Table 1). Most respondents either did not know a PCP could treat people with an opioid use disorder (OUD) by prescribing MOUD (61.4% weighted) or incorrectly believed a PCP could not (13.3% weighted). Most respondents agreed (658 [53.9%]) or strongly agreed (309 [24.9%]) that a PCP office should be a place where people can receive OUD treatment (Table 2).

**Table 1.** Association of Demographic Characteristics With Knowledge and Beliefs About MOUD and OUD Treatment in Primary Care Settings

Survey item	Participants, No. (unweighted %)	Weighted %	To the best of your knowledge, can PCPs treat people with an OUD by prescribing them a medication for their disorder? (n = 1234)				The office of a PCP should be a place where people can receive OUD treatment (n = 1221)		
			Yes, weighted %	No, weighted %	Do not know, weighted %	*P* value	Disagree, weighted %	Agree, weighted %	*P* value
Total^a^	1234 (100.0)	100.0	25.3	13.3	61.4	NA	23.8	76.2	NA
Age, y^b^									
18-29	196 (16.1)	19.7	18.4	23.8	57.8	.002	19.1	80.9	.03
30-44	354 (29.0)	25.8	28.3	10.7	60.9		17.1	82.9	
45-59	261 (21.4)	23.8	23.7	13.0	63.3		28.3	71.8	
≥60	410 (33.6)	30.7	29.3	8.7	62.0		26.6	73.4	
Gender^c^									
Male	539 (43.5)	49.1	29.1	12.7	58.2	.10	22.1	77.9	.54
Female	700 (56.5)	50.9	22.1	13.8	64.1		24.0	76.0	
Race and ethnicity^d^									
Black	143 (11.5)	12.0	27.5	19.5	53.1	.05	20.9	79.1	.62
Hispanic or Latino	188 (15.2)	17.3	18.8	19.3	62.0		26.4	73.6	
White	848 (68.4)	61.6	26.6	9.7	63.7		21.9	78.1	
Other or ≥2 races^e^	60 (4.8)	9.1	28.4	17.6	54.0		27.4	72.6	
Political party affiliation									
Democrat	429 (34.9)	33.1	25.5	16.2	58.3	.59	19.8	80.2	.03
Lean Democrat	146 (11.9)	10.6	27.3	10.2	62.5		13.3	86.7	
Lean Independent or none	217 (17.7)	19.2	25.3	11.6	63.0		30.2	69.8	
Lean Republican	112 (9.1)	9.7	17.6	13.9	68.5		25.8	74.2	
Republican	325 (26.4)	27.4	27.4	12.0	60.6		25.5	74.5	
Educational level									
Less than high school	62 (5.0)	8.8	23.7	16.8	59.6	.26	25.1	74.9	.84
High school diploma or equivalency	231 (18.6)	29.1	24.3	15.9	59.7		20.1	79.9	
Some college or associate’s degree	533 (43.0)	26.4	25.8	10.5	63.7		24.2	75.8	
Bachelor’s degree	246 (19.9)	22.4	21.7	14.4	64.0		24.4	75.6	
Postgraduate study or professional degree	167 (13.5)	13.4	35.4	8.5	56.1		23.7	76.4	

Abbreviations: MOUD, medications for opioid use disorder; NA, not applicable; OUD, opioid use disorder; PCP, primary care physicians.

^a^ In cases where a respondent skipped a question online, it was coded as missing and analyzed using complete case analysis.

^b^ Age was recorded categorically in the dataset used for analysis to protect the confidentiality of respondents.

^c^ Gender was classified as a binary for these analyses to protect the identity of nonbinary respondents, whose individual cell values were fewer than 10.

^d^ Race and ethnicity were self-identified by survey participants.

^e^ Other is defined here as self-identified race not displayed in the table, such as Asian and American Indian or Alaska Native.

**Table 2.** Knowledge, Attitudes, and Beliefs About Medications for Opioid Use Disorder in the Primary Care Setting Among a Nationally Representative Sample of US Adults^**a**^

Survey item	Participants, No. (unweighted %)	Weighted %
**Respondent has a PCP (n = 1232)^b^**		
Yes	1038 (84.3)	84.8
No	194 (15.8)	15.2
**If yes, visited PCP in past 12 mos (n = 1037)^c^**		
Yes	889 (85.7)	85.1
No	148 (14.3)	14.9
**Can PCPs treat a patient using MOUD? (n = 1234)^b^**		
Yes	327 (26.5)	25.3
No	146 (11.8)	13.3
I don’t know	761 (61.7)	61.4
**PCPs should treat OUD in their offices (n = 1221)^b^**		
Strongly agree	304 (24.9)	24.2
Agree	658 (53.9)	52.8
Disagree	212 (17.4)	19.0
Strongly disagree	47 (3.9)	4.1
**Comfort of respondents with any history of opioid misuse personally seeking MOUD from their PCP (n = 266)** ^**d**^ **^,^ ^e^**		
Very comfortable	127 (47.7)	50.6
Somewhat comfortable	89 (33.5)	30.7
Somewhat uncomfortable	28 (10.5)	10.5
Very uncomfortable	22 (8.3)	8.3
**Comfort of respondents with no history of opioid misuse referring others to their PCP for MOUD (n = 945)** ^e^ **^,^** ^f^		
Very comfortable	316 (33.4)	31.9
Somewhat comfortable	388 (41.1)	42.0
Somewhat uncomfortable	168 (17.8)	18.5
Very uncomfortable	73 (7.7)	7.7

Abbreviations: MOUD, medications for opioid use disorder; OUD, opioid use disorder; PCP, primary care physicians.

^a^ Data were collected from a June 2023 probability-based, nationally representative online and telephone survey of 1234 US adults at least 18 years of age conducted by the National Opinion Research Center at the University of Chicago. Respondents who skipped questions online had that response coded as missing. The precise wording of items and the instrument’s definitions, skip logic, and branch logic are presented in the eAppendix in Supplement 1.

^b^ All respondents received this item.

^c^ Only respondents who reported having a PCP received this item.

^d^ Only respondents who reported prior misuse of opioids received this item.

^e^ If a patient reported not having a PCP, they were asked to think of the last physician they visited for a routine checkup.

^f^ Only respondents who never reported prior misuse of opioids received this item.

Among respondents who reported ever misusing prescription or illicit opioids, 50.6% (weighted) said they would be very comfortable and 30.7% (weighted) would be somewhat comfortable personally seeking MOUD from their PCP. Of the respondents with no history of opioid misuse, 31.9% (weighted) said they would be very comfortable and 42.0% (weighted) would be somewhat comfortable referring someone they cared about to their PCP for MOUD (Table 2). In aggregate, Black respondents (20.9% weighted) were most likely to believe they could not receive MOUD at a PCP (Table 1).

## Discussion

The findings suggest most respondents did not know a PCP can provide MOUD. Raising awareness that PCPs can is critical to increasing effective treatment of OUD and reducing the race-and-ethnicity–based disparities in knowledge about MOUD access observed in this study. Interventions could include messaging campaigns similar to those for HIV testing and cancer screening. Literature and signage about MOUD could be placed in waiting areas and examination rooms. PCPs could proactively screen patients for OUD and offer to prescribe MOUD as indicated. Measures to raise awareness about the opportunity to receive MOUD from PCPs may increase demand and incentivize PCPs to offer MOUD, especially if accompanied by clinical and administrative support, such as access to addiction medicine consultations.^6^

Limitations are inherent in probability-based weighted samples and respondents’ understanding of MOUD. However, our methods found the approximately 209 000 PCPs in the US positioned to play a decisive role in expanding access to buprenorphine. Increasing the public’s knowledge of this potential may help convert it to action.

## Notes

Supplement 1.
eAppendix.

Supplement 2.
Data Sharing Statement
