# Supplementary material for: Knowledge, Attitudes, and Beliefs About Opioid Use Disorder Treatment in Primary Care
Source: JAMA Netw Open. 2024 Jun 28;7(6):e2419094. doi: 10.1001/jamanetworkopen.2024.19094 (PMC11214109; doi:10.1001/jamanetworkopen.2024.19094)
Supplement: Supplement 1. — eAppendix. [file jamanetwopen-e2419094-s001.pdf]

Supplemental Online Content

del Pozo B, Park JN, Taylor BG, et al. Knowledge, attitudes, and beliefs about opioid use disorder treatment in primary care. *JAMA Netw Open*. 2024;7(6):e2419094. doi:10.1001/jamanetworkopen.2024.19094

eAppendix.

This supplemental material has been provided by the authors to give readers additional information about their work.

© 2024 del Pozo B et al. *JAMA Network Open*

eAppendix

Methodological notes

Participants were drawn from the AmeriSpeak survey, operated by the National Opinion Research Center (NORC). Conducted since 2015 on behalf of governmental agencies, academic researchers, and media and commercial organizations, AmeriSpeak is a probability-based panel survey designed to be representative of the U.S. household population. Randomly selected U.S. households were sampled using area probability and address-based sampling, with a known, nonzero probability of selection from the NORC National Sample Frame. Households were contacted primarily by U.S. mail and telephone. The AmeriSpeak panel covers approximately 97% of the U.S. household population. Surveys were completed online, or by telephone for households without internet.

As part of AmeriSpeak's monthly survey program, randomly selected eligible panel members were invited to participate in a survey. Informed consent was obtained from all respondents: orally when participating by phone, and by electronic means when the survey was administered via an internet connection. AmeriSpeak is an ongoing survey platform designed to collect data on opinions, attitudes, and behaviors of the U.S. population, often of a sensitive nature (e.g., drug use and crime victimization). Respondents who did not respond to the initial invitation to participate were contacted over multiple rounds by phone and email, up to about six times. Participants had self-categorized their race and gender upon initial enrollment in the panel using a range of responses provided by NORC at the time, and this data is automatically associated with their response record whenever they participate in an AmeriSpeak survey. Race data were collected in the interest of understanding how the knowledge, attitudes, beliefs, and conditions investigated by Amerispeak vary by the nation's demographic groups. Participants were offered the cash equivalent of $5-7 for participation.

Statistical weights: Data were weighted to US census benchmarks, accounting for demographic selection probabilities and nonresponse. We weighted the survey data to better represent the overall US population, using US Census data. Panel-based sampling weights for all sampled housing units were computed as the inverse of the probability of selection from the NORC National Frame (the sampling frame used to sample housing units for AmeriSpeak). The study sampling weights were then derived using a combination of the final panel weight used in

© 2024 del Pozo B et al. *JAMA Network Open*

all studies using the AmeriSpeak platform and the probability of selection associated with the sampled panel member into the specific survey on opioids utilized in this study.

With many panel members not responding to our survey request (resulting in a 37% response rate), we also made a statistical adjustment for survey non-response. In analyses of respondents and nonrespondents, we found no differences in most of the demographic variables available for participants and non-participants, such as age, biological sex at birth, and race/ethnicity. We did observe small but statistically significant differences in age and geographic region, in that respondents tended to be older and from the Midwest compared to non-respondents. As discussed below, this difference was adjusted with nonresponse weights and incorporated into the final formulas used to weight the sample data.

The panel utilized a standard, validated approach to non-response weights, following the methods presented by Bethlehem, et al. in their *Handbook of Nonresponse in Household Surveys.*[1] In doing so, our approach decreases the potential nonresponse biases associated with sampled panel members who did not complete the survey interview for the study. The nonresponse adjusted survey weights for the study were adjusted via a raking ratio method to population totals for the general population aged 18 and older, associated with the following topline socio-demographic characteristics: age, sex, education, race/Hispanic ethnicity, and Census Division, household tenure, household phone status, and the following socio-demographic interactions: age x gender, age x race/ethnicity, and race/ethnicity x gender. The weights adjusted to the external population totals are the final study weights. At the final stage of weighting, any extreme weights were trimmed based on a criterion of minimizing the mean squared error associated with key survey estimates, and weights were then re-raked to the same population totals. Raking and re-raking is performed during the weighting process such that the weighted demographic distribution of survey completions resembles the demographic distribution of the target population. By aligning survey respondent demographics with the target population, the key survey items thereby align with the target population.

1. Bethlehem J, Cobben F, Schouten B. *Handbook of Nonresponse in Household Surveys*. John Wiley & Sons; 2011.

© 2024 del Pozo B et al. *JAMA Network Open*

Survey items (see branch logic predicate item below)

Q23. (Adapted from Medical Expenditure Panel Survey) Do you have a primary care provider – that is, a doctor, nurse practitioner, or other health professional that you usually go to for checkups, if you are sick, or if you need advice about your health?

RESPONSE OPTIONS:

1. Yes
2. No
3. More than one place

[Skip logic: SHOW ONLY IF Q23 = 1,3]

Q23A. During the past 12 months, did you visit your primary care provider for any reason?

RESPONSE OPTIONS:

1. Yes
2. No

Q24. To the best of your knowledge, can primary care physicians treat people with an opioid use disorder by prescribing them a medication for their disorder?

[PROGRAMMING: ADD HOVER TEXT opioid use disorder "a problematic pattern of opioid use leading to clinically significant impairment or distress."]

RESPONSE OPTIONS:

1. Yes
2. No

77. I don't know

[Branch logic: SHOW ONLY IF Q5=3]

Q24A. Think of your own primary care physician, if you have one, or otherwise think of the last doctor you went to for a routine checkup. If somebody you cared about had an opioid use disorder, would you feel comfortable referring them to that doctor for treatment for opioid use disorder?

[PROGRAMMING: ADD HOVER TEXT opioid use disorder "a problematic pattern of opioid use leading to clinically significant impairment or distress."]

RESPONSE OPTIONS:

1. Very comfortable
2. Somewhat comfortable
3. Somewhat uncomfortable
4. Very uncomfortable

[Branch logic: SHOW ONLY IF Q5=1,2]

Q24B. Think of your own primary care physician, if you have one, or otherwise think of the last doctor you went to for a routine checkup. If you were seeking treatment for an opioid use disorder, would you feel comfortable seeking treatment from that doctor?

© 2024 del Pozo B et al. *JAMA Network Open*

[PROGRAMMING: ADD HOVER TEXT opioid use disorder "a problematic pattern of opioid use leading to clinically significant impairment or distress."]

RESPONSE OPTIONS:

1. Very comfortable
2. Somewhat comfortable
3. Somewhat uncomfortable
4. Very uncomfortable

Q25. Please rate how much you agree or disagree with the following statement: The office of a primary care physician should be a place where people can receive treatment for an opioid use disorder.

[PROGRAMMING: ADD HOVER TEXT opioid use disorder "a problematic pattern of opioid use leading to clinically significant impairment or distress."]

RESPONSE OPTIONS:

1. Strongly agree
2. Agree
3. Disagree
4. Strongly disagree

Branch logic predicate item

Q5. – Sets the branch logic for questions 24a and 24b:

Have you ever taken a prescription opioid medication (e.g., fentanyl, oxycodone [OxyContin, Percocet], hydrocodone [Vicodin], methadone, buprenorphine, etc.) in a way other than as directed by a medical provider or non-prescribed/illegal opioids, for example, to feel good/get high or because you would get sick if you didn't take it?

RESPONSE OPTIONS:

1. Yes, within the last year
2. Yes, more than a year ago
3. No.

© 2024 del Pozo B et al. *JAMA Network Open*
