# Supplementary material for: Knowledge, Attitudes, and Beliefs About Opioid Use Disorder Treatment in Primary Care
Source: JAMA Netw Open. 2024 Jun 28;7(6):e2419094. doi: 10.1001/jamanetworkopen.2024.19094 (PMC11214109; doi:10.1001/jamanetworkopen.2024.19094)
Supplement: Supplement 2. — Data Sharing Statement [file jamanetwopen-e2419094-s002.pdf]

Data Sharing Statement

del Pozo. Knowledge, Attitudes, and Beliefs About Opioid Use Disorder Treatment in Primary Care. *JAMA Netw Open*. Published June 28, 2024. doi:10.1001/jamanetworkopen.2024.19094

Data
Data available: Yes
Data types: Deidentified participant data
How to access data: Please email Dr. Bruce Taylor at taylor-bruce@norc.org to request the data.
When available: With publication

Supporting Documents
Document types: None

Additional Information
Who can access the data: Anyone requesting.
Types of analyses: Any purpose.
Mechanisms of data availability: Without support.
Any additional restrictions: None.
